# MedHerent: Improving Medication Adherence in Older Adults With Contextually Sensitive Alerts Through an Application That Adheres to You

**DOI:** 10.1016/j.mcpdig.2023.11.001

**Published:** 2023-12-14

**Authors:** Andrew Nguyen, Saumya Uppal, Mikaela Mendoza Pereira, Andreea Pluti, Lisa Gualtieri

**Affiliations:** Tufts University School of Medicine, Boston, MA

## Abstract

Medication adherence has long been viewed as a patient issue, but what if we shift this perspective? What if medications could adjust to the needs and context of patients, instead of the other way around? We used design thinking to create a contextually sensitive digital health mobile application to improve medication adherence in older adults. We define contextual sensitivity as sensitivity to the context of patient needs. Through persona and scenario ideation, interviews, evaluations of existing solutions, prototypes, and consultations with subject matter experts, we uncovered key barriers to medication adherence. We outline 4 key challenges: alert fatigue, poor health literacy, lack of social support, and lack of behavior change and motivation, which are specific to older adults. The resulting application features reminders and alerts, a dashboard and calendar, educational resources, social sharing, and reward features. These 5 elements emphasize the significance of design thinking, contextual sensitivity, trimodal alerts, and co-interventions in developing effective digital health solutions for medication adherence among older adults.

About 50% of older adults struggle with medication adherence. Chronic conditions, complicated prescriptions, and incorrect dosages may lead to worsening health conditions, costs, and morbidity and mortality rates.^1^, ^2^, ^3^ Digital health solutions may offer cost effective and accessible ways to improve adherence and patient outcomes.^4^, ^5^, ^6^, ^7^, ^8^ However, they may also introduce new challenges.^9^ For instance, some older adults struggle with adjusting the settings of mobile applications (apps) and find reminders too distracting.^10^ Despite these barriers, many older adults (83%) are eager to learn how to improve their physical and mental well-being.^11^ A medication adherence app designed with the specific needs of older adults in mind may provide valuable insights into how technology can help close healthcare gaps.^12^

Although pill boxes may improve patient satisfaction and outcomes by providing reminders, they have not yet reported adherence benefits compared with traditional bottles or electronic versions for complicated regimens.^13^, ^14^, ^15^ Other approaches, such as health literacy tools, reminders, and digital social support are currently being investigated.^9^^,^^16^

Health literacy tools, like digital flashcards and videos, may improve medication adherence in older adults when used with adherence solutions.^17^ However, poor comprehension may hinder compliance.^1^^,^^18^ Personalized, interactive, and action-oriented health literacy tools have been shown to be effective, but more research is needed to assess its impact on adherence for older adults.^19^^,^^20^

Social and professional support may also enhance medication adherence. For example, improvements were shown with caregiver support from the Medisafe app.^21^ An app with nursing coach support also improved adherence, outcomes, and satisfaction.^22^ Social support can not only benefit adherence, but also mental health, stress, and sleep.^23^ More research is needed to explore the impact of support on medication adherence for older adults.^24^

Single-mode interventions, like automated phone calls, showed no impact on adherence for oral oncogenic drugs, whereas SMS reminders improved adherence for patients with acute coronary syndrome.^17^^,^^25^ Meanwhile, combined text and voice messaging, and multiple smartphone-based interventions improved outcomes.^26^^,^^27^ Another trial found that text, phone, and email reminders resulted in increased adherence, but further research is needed to assess how multimodal alerts may impact medication adherence for older adults.^20^

Interventions that take into account the unique needs and experiences of patients may be more effective. Health literacy tools that use simple language and personalized content to meet the demographic characteristics, behavioral, and psychological needs of patients have been shown to improve medication adherence.^18^^,^^19^ Social support from friends and family, and professional support from health care providers, can also lead to better clinical outcomes and higher levels of patient satisfaction.^22^ Flexible reminders, such as those using multiple modes of delivery (text message, phone, or email), have also been shown to considerably improve medication adherence.^23^

As a result, sensitivity to the context of patient needs, a term we define as contextual sensitivity, is a crucial aspect of digital health solutions aimed at improving medication adherence in older adults. Despite encouraging findings, the role of contextual sensitivity in digital health apps for medication adherence in older adults is not well documented. The rationale of this study is to explore how contextual sensitivity may be implemented in digital health apps for medication adherence in older adults.

In collaboration with the American Association of Retired Persons (AARP) and Tufts University School of Medicine faculty as part of a semester-long graduate level digital health course, we were tasked to use design thinking to create an app to address barriers in contextual sensitivity for older adults. Design thinking is a systematic and iterative process that involves the desires, needs, and limitations of patients.^28^^,^^29^ The stages of design thinking involve understanding the problem, defining the target population, identifying their needs, ideating innovative solutions, experimenting, and refining solutions through feedback.^30^ Kolnick et al^31^ successfully used design thinking in a telehealth project for older adults, finding that multiple perspectives and ideations helped overcome barriers and implement crucial features. Using design thinking, our interdisciplinary team developed MedHerent, a digital health medication adherence app for older adults.

## Methods

In the design thinking process, we created personas and scenarios, performed interviews, evaluated existing solutions, developed prototypes, and consulted subject matter experts (SMEs). We started by deeply understanding the needs and problems facing our target personas—older adults and their caregivers—through immersive personas and interviews. This guided the ideation process, where we brainstormed a wide range of solutions. Prototypes were then created and iteratively refined, using the feedback from the interviews to drive design decisions. Our strengths, weaknesses, opportunities, and threats (SWOT) analysis of existing solutions further informed the design process, allowing us to identify gaps and opportunities in the market. This cyclical, user-centric approach is fundamental to design thinking and was integral in shaping our study’s design and implementation.

First, we developed primary and secondary personas representing older adults who could benefit from our solution. Scenarios were created for each persona, outlining their experience and perspective on medication adherence. Then, we conducted interviews with participants resembling our primary and secondary personas to gain insights on the daily lives of older adults, their perspectives on medication adherence, and gaps in their health needs.

Our semi-structured interviews were hosted through video conference, focusing on 2 distinct groups: adults aged 65 years and older, and those who care for them. All participants, sourced from a convenience sample of friends and family, were actively involved with medication use and provided verbal consent. Each interview was facilitated by 2 team members: one leading the discussion on medication adherence and the other meticulously documenting responses. After consolidating the interview notes, we collectively analyzed the data to identify recurring themes and gaps in medication adherence. This analysis allowed us to prioritize previously overlooked needs, thereby refining our app to better serve older adults. We did not seek IRB approval or waiver for this class project for educational purposes.

Second, we evaluated existing digital solutions for medication adherence in older adults by conducting SWOT analyses. On the basis of our literature review, personas, interviews, and analysis of existing solutions, we developed an app prototype using Figma, a collaborative web app for interface design. Ideation of the step-by-step processes that patients take to adhere to their medications, also known as journey maps, helped us validate, test, and address missing functionalities. Finally, we sought feedback from SMEs from AARP and Tufts University School of Medicine faculty to refine our app. We made new iterations of our app as recommended. The length of the study was 1 semester from January 2022 to May 2022.

## Results

We developed 3 primary personas, Deborah, Miguel, and André, and 1 secondary persona, Meegan. Primary personas were created to represent our main target patient group, older adults, whereas a secondary persona was designed to represent their caregivers. Although a secondary persona is not the main focus, both persona types are central to the design thinking process because medication adherence for older adults involves many stakeholders.

Deborah, a 60-year-old single woman, seeks a patient-friendly, interactive calendar with alerts to manage medications on her own. Miguel, a 50-year-old Hispanic male, desires an app that improves PrEP adherence and integrates social features. André, a 52-year-old African American man, aims to self-manage his type 2 diabetes treatment and seeks a culturally sensitive, easy-to-understand medication guide. Meegan, André’s wife and caregiver, wants an app that empowers her husband through sharing functions, motivation, and gamification to maintain medication adherence. Full patient personas are summarized in Appendix A.

To gather insights, we conducted semi-structured interviews with 4 participants (n=4) representing our personas (Table 1). All participants were friends and family members who responded positively to our request. In total, we approached 4 participants (n=4) for a 100% response rate.

**Table 1:** List of Interview Questions

1. Can you run me through what your daily life looks like?
2. Tell me about the medications you take and how often do you have to take them?
3. How would you describe your digital health literacy? How proficient are you with digital technology?
4. How would you describe your adherence to your medications? a) If poor, why? Could you be more detailed?
5. What is one thing you would take out of your medication regimen if you could?
6. How do you perceive your medications? a) Is your medication a need or an annoyance/negative reminder?
7. How willing would you be to utilize an app to facilitate your medication regimen? a) Why or why not?
8. What do you see as the biggest challenges in adhering to medications?
9. What solutions do you think would work best to help you adhere to your medications?
10. What do you think healthcare should consider more when thinking about ways to improve medication adherence?

Participant 1 sought to improve their medication adherence, was technology-savvy, and had a positive outlook toward medications. They expressed interest in using an app to help keep track of their medications, with features such as notifications and medication summaries. Participant 2 led a busy and active lifestyle and was proficient with technology. Although maintaining a consistent medication regimen, they occasionally missed taking a multivitamin. They were keen to use an app to manage medications and were interested in educational features that would help them understand their regimen. Participant 3, facing multiple health challenges, had a negative perception of medications because of side effects and preferred personal reminders for medication adherence rather than using an app. Participant 4, a caregiver, found app features like alarms, tracking, video walkthroughs, and caregiver monitors beneficial for managing their family member’s medications. Full responses are summarized in Appendix A.

The interviews offered important insights into our target audience’s lifestyles, medication adherence needs, and technological preferences, guiding further research and development. We found that most interviewees were open to using an app for medication adherence, behavior change maintenance was the main challenge, and patient motivation and experience were crucial factors in our initiative’s development. These findings greatly influenced our project by helping us identify patient needs, select app features, and stress the importance of behavior change.

Our SWOT analysis of existing solutions, including generic pill cards, provided insights for our initiative.^32^, ^33^, ^34^, ^35^, ^36^ Generic pill cards are low-cost and intuitive but lack the flexibility and integration needed for older adults. GenXys offers personalized medication support but may be too complex for those with limited health and digital literacy. MediSafe, a medication engagement app, provides behavior-based interventions and coaching but assumes a certain level of health literacy. Pavlok, a wearable device, uses aversive conditioning to build habits, but not all older adults may appreciate its approach. Dose Health, a portable medication management device, offers multimodal reminders but may be expensive. A full table for our SWOT analysis for each solution is summarized in Appendix A.

Our personas, interviews, and SWOT analysis validated 4 core unmet needs in medication adherence for older adults: alert fatigue, poor health literacy, lack of social support, and lack of behavior change and motivation (Table 2).

**Table 2:** Unmet Needs for Older Adults and MedHerent’s Approach to Bridging Them

Unmet Need	MedHerent Features
Alert fatigue	Contextually sensitive alerts, dashboard, and calendar
Poor health literacy	Medication dictionary
Lack of social support	Friends integration
Lack of behavior change and motivation	Rewards system

From our evaluation of personas, scenarios, interviews, and existing solutions, we formulated a final prototype with 5 features to fulfill identified unmet needs: contextually sensitive alerts, a flexible dashboard and calendar, a medication dictionary, a friend’s integration, and a rewards system (Appendix B). The dashboard and calendar helped Deborah self-manage her medications after her husband’s passing, while André improved his health literacy through the accessible medication dictionary. Miguel connected with a support network using the interactive social feature, and Meegan used the rewards function to stay motivated while assisting André.

Contextual alerts combined with a status dashboard and a long-term calendar are proposed to mitigate alert fatigue, presenting multiple pathways to monitor both short-term and long-term adherence. According to our interviews, contextual sensitivity may do more than just enhance adherence, it may also boost patient satisfaction. Older adults reported a preference to assess their medication adherence progress over time, underscoring the dashboard and calendar’s pivotal role in promoting self-efficacy and self-management.

Next, the medication dictionary aims to bridge health literacy gaps. By offering a searchable platform comprising details on medications, dosages, and appropriate usage, it goes beyond mere listing. The dictionary renders medication regimens in context with supplementary health particulars, empowering patients to steer their health narratives beyond the app’s boundaries.

Meanwhile, the opt-in friends feature addresses the need for social support in medication adherence. MedHerent’s social integration facilitates a community space for older adults, letting them bridge connections with peers confronting analogous health challenges. This feature serves as a platform for sharing experiences, milestones, and adherence streaks, with the option for patients to regulate the visibility of their progress.

Finally, the rewards system intertwines intrinsic and extrinsic motivational facets. By engaging with the app, patients accrue points, which they can subsequently redeem for health-focused subscriptions. This reward infrastructure not only celebrates their consistent adherence endeavors but also fosters a sustained behavior change. MedHerent’s overarching objective is to provide a comprehensive and contextually sensitive method to medication adherence, empowering patients to become stewards of their health.

MedHerent’s development was guided by SME feedback, leading to adjustments in our personas, research into unmet market needs, and addressing concerns about patient data, education, privacy, and health care regulations. The SMEs suggested partnering with pharmacies for in-app revenue, which prompted further exploration of partnerships with physicians and practices. In response, we updated the app to include electronic health record synchronization, added an option for inputting personal information, refined the medication dictionary with trivia notifications, and investigated physician alerts subject to regulations.

## Discussion

We validate the efficacy of the design thinking process in crafting digital health apps to enhance medication adherence among older adults. The process guided us to create app features tailored to their unique needs. We observed that older adults may benefit from more than just a simple medication adherence app: they seek multimodal alerts, educational tools, social empowerment, and long-term behavior change. Without constant patient-driven insights and feedback, we might have overlooked these crucial needs and features.

Beyond a novel application of these features for medication adherence in older adults, our study is also the only one of its kind to explore the role of contextual sensitivity. Contextual sensitivity, in this study, refers to understanding the unique environments in which older adults live and grow. Recognizing that different demographic characteristics have distinct needs, our use of design thinking specifically unearthed the medication adherence challenges faced by older adults. Informed by this understanding, we crafted an app steeped in contextual awareness. Such sensitivity provides a tailored, patient-centric approach, ensuring that health care solutions meet the specific requirements of their intended audience. Indeed, medication adherence presents a 2-way challenge between patients and healthcare providers. Contextual sensitivity challenges the traditional view that this is solely a patient-centered issue.

Trimodal alerts through phone calls, app notifications, and text messages are a core component of contextual sensitivity. Journey maps and interviews reveal that using a variety of alert methods may decrease alert fatigue and enhance patient satisfaction. Future work could explore how these alert types affect adherence. The specific content of each alert also needs further exploration. Addressing challenges in basic medication regimens may also minimize patient frustration with the app, aiming to boost medication adherence among older adults.

Combining multiple co-interventions in tandem with trimodal alerts may also promote medication adherence in older adults. The dashboard and calendar features, for example, enable older adults to visually track their medication-taking habits. A streak of consistent medication adherence may instill a sense of accomplishment, while noting missed doses can serve as a reminder to get back on track. Meanwhile, the rewards system offers both intrinsic rewards, like the satisfaction of maintaining good health, and extrinsic rewards, which can be tangible benefits or recognitions. Metrics such as adherence rates and reward scores not only encourage regular medication intake but also offer positive reinforcement. When used together, these interventions complement one another, creating a feedback loop, or habit stack, that may encourage behavior change and sustain adherence.

MedHerent, developed during a graduate digital health course, has several limitations. The project requires further market research, a larger and more diverse interview sample, and deeper exploration of development challenges. To ensure the app’s success in the health care market, essential steps include securing funding, leveraging best practices from organizations like AARP, initiating pilot projects, and gathering feedback from focus groups and surveys. Engaging with experts in health care app design and conducting extensive patient testing may refine our design. Further refinements may also address medication adherence efficacy, app deliverability, and payor costs. The limited interview sample of 4 individuals, selected by convenience sampling, may also be expanded for more comprehensive insights. The sample size was too small to conduct statistical analyses.

## Conclusion

We propose a digital health solution for older adults to improve their medication adherence, called MedHerent. This app challenges the conventional definition of non-adherence by using design thinking to create contextually sensitive features that meet the unique needs of older adults. The 5 core functions, including reminders and alerts, a dashboard and calendar, educational resources, social sharing, and reward features, create a 2-way adherence relationship between patients and providers. Beyond contextual sensitivity, we show how trimodal alerts and co-interventions may be more comprehensive and effective than singular interventions. We conclude that design thinking is an effective approach to create comprehensive, empathetic, and patient-centered health care apps.

## Potential Competing Interests

The authors declare no potential conflicts of interest with respect to the research, authorship, or publication of this article.

## References

[1] Evans L., Spelman M. (1983). The problem of non-compliance with drug therapy. Drugs.

[2] Klein D (2021). https://www.panfoundation.org/medication-non-adherence/.

[3] Bunis B.D. (2019). High prescription drug prices lead many consumers to ignore doctors’ orders. AARP.

[4] Jandoo T. (2020). WHO guidance for digital health: what it means for researchers. Digit Health.

[5] Gandapur Y., Kianoush S., Kelli H.M. (2016). The role of mhealth for improving medication adherence in patients with cardiovascular disease: a systematic review. Eur Heart J Qual Care Clin Outcomes.

[6] Hamine S., Gerth-Guyette E., Faulx D., Green B.B., Ginsburg A.S. (2015). Impact of mhealth chronic disease management on treatment adherence and patient outcomes: a systematic review. J Med Internet Res.

[7] Anglada-Martinez H., Riu-Viladoms G., Martin-Conde M., Rovira-Illamola M., Sotoca-Momblona J.M., Codina-Jane C. (2015). Does mhealth increase adherence to medication? Results of a systematic review. Int J Clin Pract.

[8] Davidson H.E. (2016). When the pill box isn’t enough. Consult Pharm.

[9] Scott Kruse C., Karem P., Shifflett K., Vegi L., Ravi K., Brooks M. (2018). Evaluating barriers to adopting telemedicine worldwide: a systematic review. J Telemed Telecare.

[10] Huang Z., Tan E., Lum E., Sloot P., Boehm B.O., Car J. (2019). A smartphone app to improve medication adherence in patients with Type 2 diabetes in Asia: feasibility randomized controlled trial. JMIR mHealth uHealth.

[11] Kakulla B. (2022).

[12] Murray M. (2004). A conceptual framework to study medication adherence in older adults. Am J Geriatr Pharmacother.

[13] Partridge A.H., Avorn J., Wang P.S., Winer E.P. (2002). Adherence to therapy with oral antineoplastic agents. J Natl Cancer Inst.

[14] MacIntosh P.W., Pond G.R., Pond B.J., Leung V.C.M., Siu L.L. (2007). A comparison of patient adherence and preference of packaging method for oral anticancer agents using conventional pill bottles versus daily pill boxes. Eur J Cancer Care (Engl).

[15] Choudhry N.K., Krumme A.A., Ercole P.M. (2017). Effect of reminder devices on medication adherence: The REMIND Randomized Clinical Trial. JAMA Intern Med.

[16] Lansberg P., Lee A., Lee Z.V., Subramaniam K., Setia S. (2018). Nonadherence to statins: individualized intervention strategies outside the pill box. Vasc Health Risk Manag.

[17] Yeung D.L., Alvarez K.S., Quinones M.E. (2017). Low–health literacy flashcards and mobile video reinforcement to improve medication adherence in patients on oral diabetes, heart failure, and hypertension medications. J Am Pharm Assoc (2003).

[18] Dunn P., Conard S. (2018). Improving health literacy in patients with chronic conditions: A call to action. Int J Cardiol.

[19] Conard S. (2019). Best practices in digital health literacy. Int J Cardiol.

[20] Xu H., Long H. (2020). The effect of smartphone app–based interventions for patients with hypertension: systematic review and meta-analysis. JMIR mHealth uHealth.

[21] Morawski K., Ghazinouri R., Krumme A. (2018). Association of a smartphone application with medication adherence and blood pressure control: the MedISAFE-BP randomized clinical trial. JAMA Intern Med.

[22] Moore J.O., Marshall M.A., Judge D.C. (2014). Technology-supported apprenticeship in the management of hypertension: A randomized controlled trial. J Clin Outcomes Manag.

[23] Thayer C., Dinger E. (2016).

[24] Chow C.K., Redfern J., Hillis G.S. (2015). Effect of lifestyle-focused text messaging on risk factor modification in patients with coronary heart disease: a Randomized Clinical Trial. JAMA.

[25] Sikorskii A., Given C.W., Given B.A. (2018). An automated intervention did not improve adherence to oral oncolytic agents while managing symptoms: results from a two-arm randomized controlled trial. J Pain Symptom Manage.

[26] Greer J.A., Jacobs J.M., Pensak N. (2020). Randomized trial of a smartphone mobile app to improve symptoms and adherence to oral therapy for cancer. J Natl Compr Canc Netw.

[27] Sheppard J.P., Tucker K.L., Davison W.J. (2020). Self-monitoring of blood pressure in patients with hypertension-related multi-morbidity: systematic review and individual patient data meta-analysis. Am J Hypertens.

[28] Roberts J.P., Fisher T.R., Trowbridge M.J., Bent C. (2016). A design thinking framework for healthcare management and innovation. Healthcare (Amsterdam, Netherlands).

[29] Design thinking: a beginner’s guide. https://www.adobe.com/creativecloud/design/discover/design-thinking.html.

[30] Cassarino N., Bergstrom B., Johannes C., Gualtieri L. (2021). Monitoring older adult blood pressure trends at home as a proxy for brain health. Online J Public Health Inform.

[31] Kolnick H.A., Miller J.E., Dupree O., Gualtieri L. (2021). Design thinking to create a remote patient monitoring platform for older adults’ homes. Online J Public Health Inform.

[32] Agency for Healthcare Research and Quality, Rockville MD. How to create a pill card. https://www.ahrq.gov/questions/resources/pill-card.html#:%7E:text=A%20pill%20card%20is%20a,follow%20a%20regular%20daily%20schedule.

[33] GenXys (2022). *Precision Prescribing Software*. https://www.genxys.com/.

[34] Medisafe (2022). *Seamlessly Manage the Medication Journey*. https://www.medisafe.com/.

[35] Pavlok. *Change Your Habits and Life with Pavlok.* 2022. https://pavlok.com/. Accessed May 3, 2022.

[36] Dose health (2020). *Dose Health*. https://www.dosehealth.com/.

