# Supplementary material for: MedHerent: Improving Medication Adherence in Older Adults With Contextually Sensitive Alerts Through an Application That Adheres to You
Source: Mayo Clin Proc Digit Health. 2023 Dec 14;2(1):1–7. doi: 10.1016/j.mcpdig.2023.11.001 (PMC11975706; doi:10.1016/j.mcpdig.2023.11.001)
Supplement: Appendix [file mmc1.pdf]

Appendix A

	Deborah	Miguel	André	Meegan
Age	60	50	53	51
Sex/Gender	Female	Male	Male	Female
Race/Ethnicity	White (Non-Hispanic/Latinx)	Hispanic	African American	Caucasian
Marital Status	Single	Single	Married to Meegan	Married to André
Education Level	Bachelor's in Art History	Master's in Food Science	High School Graduate	Bachelor's in Business
Location	Hartford, CT	San Francisco, CA	New York, NY	New York, NY
Description	Deborah, a curator in Hartford, CT, lost her husband a year ago. Her husband's death, who managed her medications, was a trigger for Deborah, affecting her adherence.	Miguel, a programmer in San Francisco, is working towards his Master's. Due to his busy life, he struggles to adhere to his HIV preventative medication.	André, a voice actor in NYC, relies on his wife Meegan to manage his type 2 diabetes medications. He possesses moderate digital literacy.	Meegan manages André's medications while running a small jewelry business. As André's caregiver, she seeks a solution to ease the responsibilities of caregiving.

Scenario	Deborah's depression worsened due to irregular medication intake, prompting intervention from her estranged daughter. She needs an alert-based calendar for medication tracking.	Miguel, with high digital literacy, requires an interactive app with a social aspect to improve his medication adherence.	André requires a culturally sensitive and user-friendly app with social and reward elements for his diabetes management.	Meegan aims to empower André in managing his medications, looking for an app to motivate him and alleviate her caregiving stress.
Quote	"It would be great if I could have a convenient and interactive calendar with alerts."	"An app that allows me to interact with friends and prioritize my medication would be a great addition to my lifestyle."	"I would like an app that's easy to use and has a social component."	"If only there was an alternative solution to reduce the stress of caregiving!"

Supplementary Table 1: This table presents a comprehensive comparison of four distinct patient personas: Deborah, Miguel, André, and Meegan. Key demographics, including age, gender, ethnicity, marital status, educational background, and location are provided. Additionally, each persona is characterized by a brief description, a representative scenario highlighting their unique challenges or needs related to medication adherence, and a quote encapsulating their primary wish or requirement from a digital solution.

	Participant 1	Participant 2	Participant 3	Participant 4
Can you run me through what your daily life looks like?	Wake up at 7:30am, work from 8am to 4:30pm, lunch from 12pm to 12:30pm, dinner from 4pm to 5:30pm, rehearsal from 6pm to 9pm, take medication at 9:30pm, sleep between 10pm and 10:30pm	Wake up at 7am, workout, work until 7pm, enjoy dinner and watch TV until 11pm	Wake up at 6:30am, work from 7:45am to 6pm, intermittent fasting from 3pm to 9pm, attend son's sports activities from 6pm to 9pm, go to sleep at midnight.	Wake up at 5am, prepare breakfast from 6am to 7am, drive child to school, engage in gardening, gym, and grocery shopping, pick up child at 2pm, watch a movie at 3pm, have dinner at 5pm, take medications after dinner
Tell me about the medications you take and how often you have to take them?	Propranolol, Abilify, Zoloft, Birth control, NSAIDs	Metformin, Multivitamin	Synthroid, Effexor, Pradaxa, Vitamins D3 & B12	Seizure medications

How would you describe your digital literacy/ how proficient are you with digital technology?	Intermediate advanced	Proficient, frequently uses digital devices	Intermediate, mostly for work	Basic, knows essentials
How would you describe your adherence to your medications?	Tries to adhere but sometimes forgets	Prioritizes Metformin over multivitamin	Good adherence, self-adjusts Synthroid occasionally	Good due to routine
Reasons for Poor Adherence (if any)	Busy schedule or fatigue	Sometimes too tired or forgetful	N/A	N/A
What is one thing you would take out of your	Less frequent intake, better knowledge	Reduce number of pills	Discontinue Pradaxa & reduce	Bulk medications with exact dates

medication regime if you could?			side-effects from Effexor	
How do you perceive your medications?	Lifesaving but requires commitment	Necessary but a reminder of illness	Lifesaving, essential post thyroidectomy	Concerns about long-term effects and dosages
Is it a need or an annoyance/negative reminder?	Both	Both	Both	Both
How willing would you be to utilize an application to facilitate your medication regime?	Willing but might ignore notifications	Very willing	Reluctant due to minimal tech use	Helpful for child's independent care

Why or why not?	Extension of current reminders, needs info on medication	Always close to phone, believes in app integration	Tends to turn off notifications, minimal weekend tech use	Monitor for child, reduce user overload
What do you see are the biggest challenges in adhering to medications?	Busy lifestyle, fatigue	Forgetting when tired or busy	Waiting post-Synthroid, busy schedule	Long-term medication effects on young age
What solutions do you think would work best to help adhere to medications?	Patient education, reminders, motivation	Reminder app, smaller medication sizes	Direct explanations, reminders, apps for cognitively impaired	Smaller doses, health maintenance, reliable app with reminders

Supplementary Table 2: The table provides an organized summary of the responses given by four interviewees in response to a series of questions about their daily lives, medication regimens, digital proficiency, perception of their medications, challenges with adherence, and views on potential solutions.

Product	Strengths	Weaknesses	Opportunities	Threats
Gener ic pill cards	Low-cost, intuitive, easy to use	Lack flexibility and integration, not suitable for older adults	Develop new features to meet the needs of older adults, integrate with other healthcare devices and services	New entrants to the market, changes in government regulations
GenX ys	Personalized medication support, comprehensive features	Too complex for those with limited health and digital literacy	Develop a simpler version for users with limited health and digital literacy, partner with healthcare organizations to reach more users	Changes in government regulations, new entrants to the market
Medi Safe	Behavior-based interventions and coaching, medication engagement app	Assumes a certain level of health literacy	Develop educational resources to improve health literacy, partner with healthcare organizations to reach more users	Changes in government regulations, new entrants to the market
Pavlo k	Uses aversive conditioning to build habits, effective for some users	Not all older adults may appreciate its approach	Develop different modes of operation to appeal to a wider range of users, partner with healthcare organizations to reach more users	Changes in government regulations, new entrants to the market
Dose Healt h	Portable medication management device, multimodal reminders	Expensive	Develop a more affordable version, partner with insurance companies to make it more accessible to users	Changes in government regulations, new entrants to the market

Supplementary Table 3: The table presents a SWOT analysis for five different existing medication management solutions: Generic pill cards, GenXys, MediSafe, Pavlok, and Dose Health. For each product, the analysis provides details on their strengths, weaknesses, opportunities, and threats. Strengths highlight the notable advantages of the products, whereas weaknesses indicate their limitations. Opportunities suggest potential improvements or strategies that could enhance the product's value proposition, while threats pinpoint external factors that could challenge the product's success in the market.

Appendix B

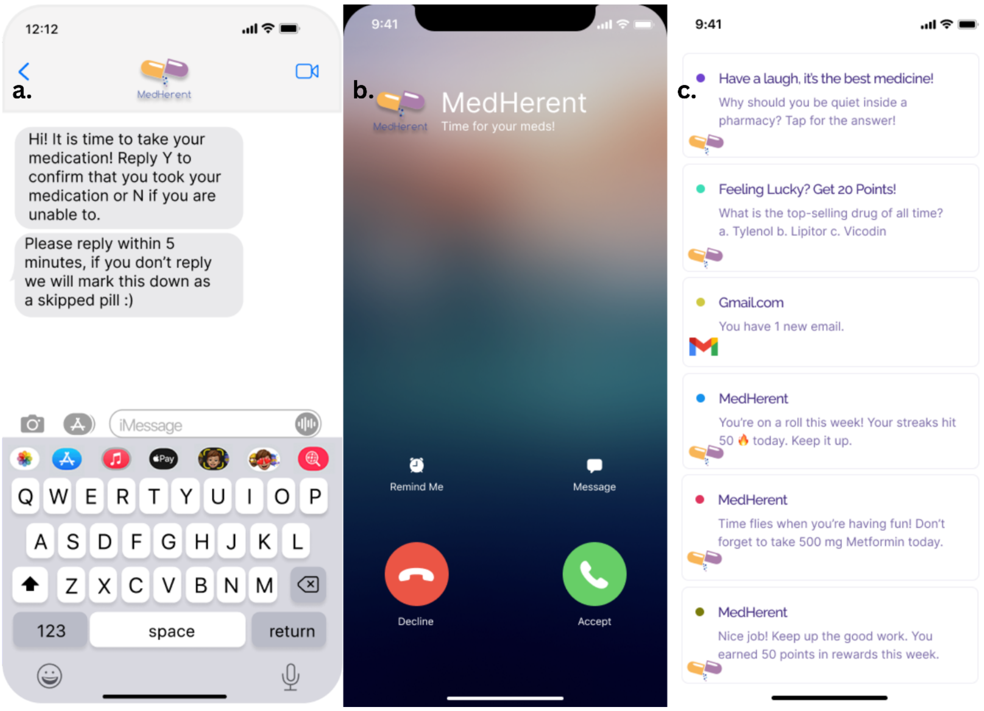

Supplementary Fig. 1: Trimodal Interaction System for Reminders. The system allows patients to receive reminders via texts (a), automated calls (b), or mobile notifications (c) to take their medications.

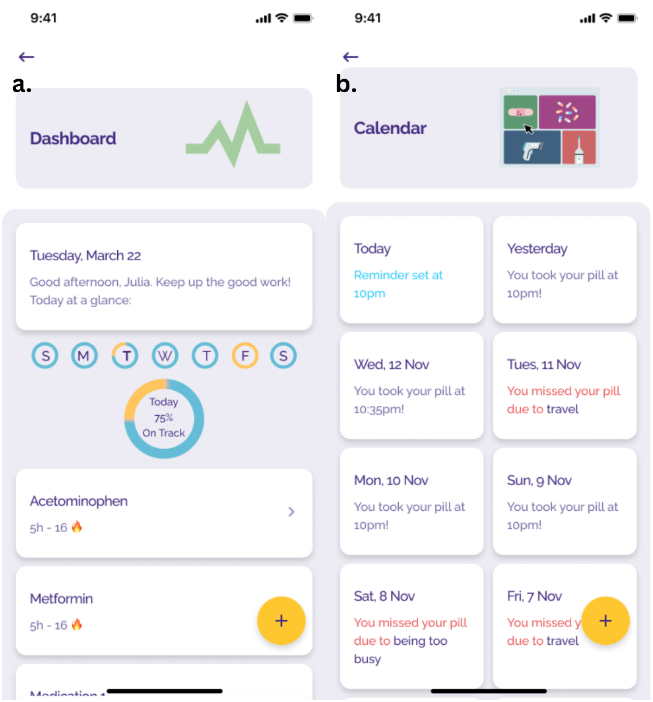

Supplementary Fig. 2: Application Dashboard and Calendar for Adherence Monitoring. The dashboard (a) displays daily adherence on a weekly scale, encouraging patients to complete their adherence rings daily, represented by a fire emoji for daily streaks. The calendar (b) provides a more detailed view of adherence over time.

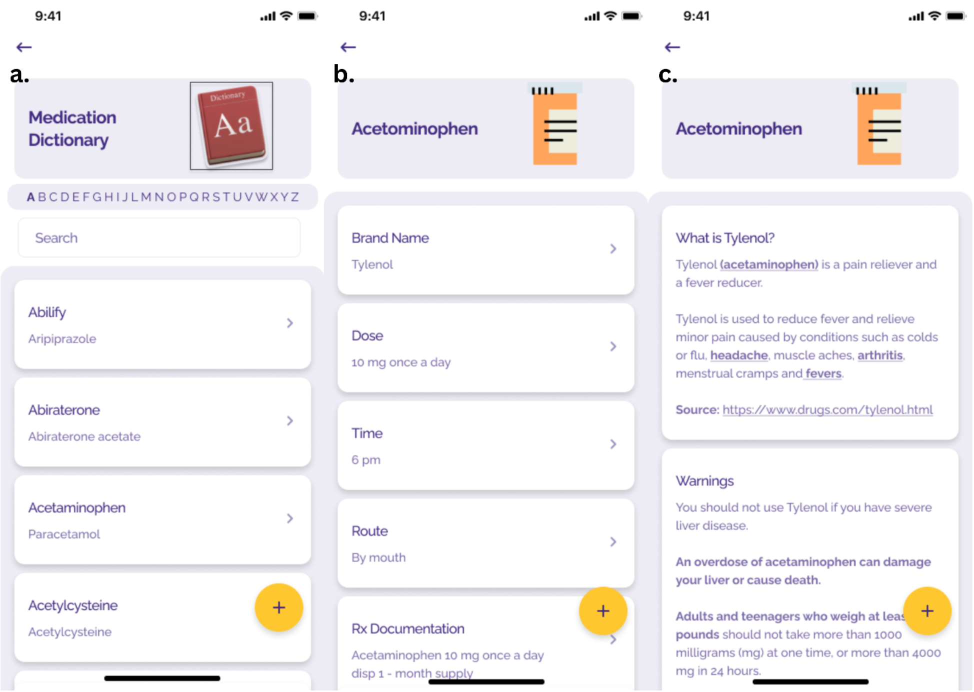

Supplementary Fig. 3: Medication Dictionary and Acetaminophen Overview within the Application. The medication dictionary allows patients to view different medications by their brand and chemical names (a). Selecting a medication reveals detailed information including brand name, dosage, timing, route, and provider documentation (b). Further tapping on 'Rx Documentation' provides an in-depth perspective on the medication, including explanations, warnings, and external resources (c).

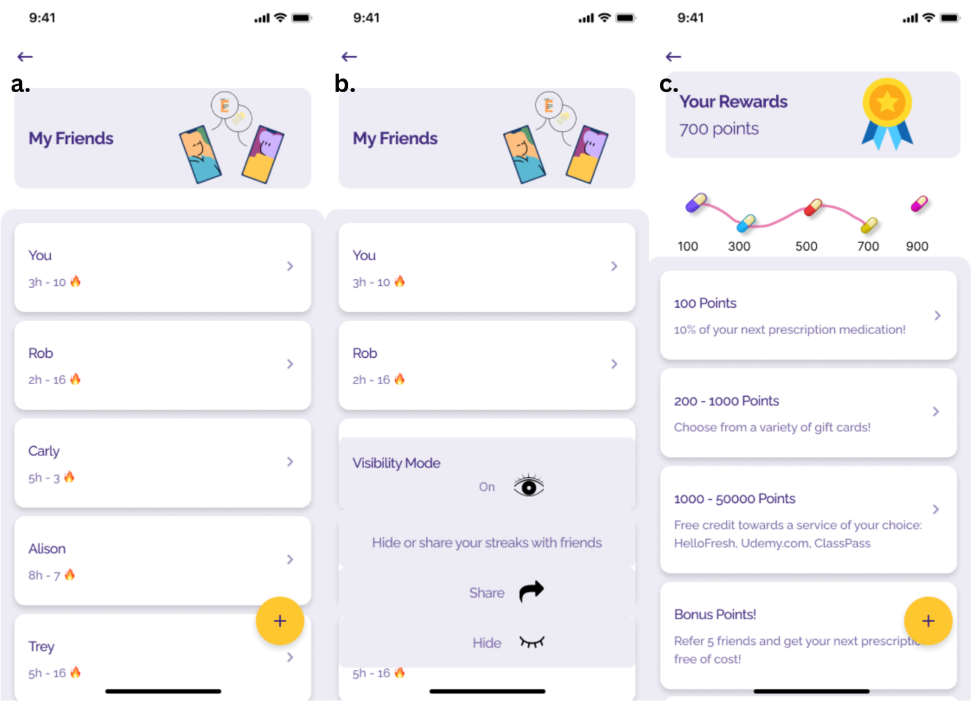

Supplementary Fig. 4: Friends and Rewards Pages. The My Friends page allows patients to interact and compare streaks with others (a), with an option to hide or share streaks while maintaining a sense of community (b). The Rewards page enables patients to redeem points for rewards or activities from partnering vendors (c).
